# Adherence to antidepressant therapy for major depressive patients in a psychiatric hospital in Thailand

**DOI:** 10.1186/1471-244X-10-64

**Published:** 2010-08-22

**Authors:** Benjamas Prukkanone, Theo Vos, Philip Burgess, Nathorn Chaiyakunapruk, Melanie Bertram

**Affiliations:** 1 School of Population Health, University of Queensland Herston, QLD 4006, Australia; 2 Faculty of Pharmaceutical Sciences, Naresuan University, Phitsanulok 65000, Thailand

## Abstract

**Background:**

Poor adherence to antidepressant therapy is an important barrier to the effective management of major depressive disorder. This study aims to quantify the adherence rate to antidepressant treatment and to determine the pattern of prescriptions of depressed patients in a psychiatric institute in Thailand.

**Methods:**

This retrospective study used electronic pharmacy data of outpatients aged 15 or older, with a new diagnosis of major depression who received at least one prescription of antidepressants between August 2005 and September 2008. The medication possession ratio (MPR) was used to measure adherence over a 6 month period.

**Results:**

1,058 were eligible for study inclusion. The overall adherence (MPR > 80%) in those attending this facility at least twice was 41% but if we assume that all patients who attended only once were non-adherent, adherence may be as low as 23%. Fluoxetine was the most commonly prescribed drug followed by TCAs. A large proportion of cases received more than one drug during one visit or was switched from one drug to another (39%).

**Conclusions:**

Adherence to antidepressant therapy for treatment of major depression in Thailand is rather low compared to results of adherence from elsewhere.

## Background

Depressive disorders are associated with significant health and social burden. In the Thai burden of disease study in 2004, it ranked as one of the top ten causes of Disability Adjusted Life Years (DALYs)[1]. Major depressive disorder is recognized as a chronic episodic disorder [2]. National treatment guidelines for major depression recommend at least six months of continuation therapy to prevent relapse and recurrence [3]. According to a review of non-adherence with antidepressant therapy, values of between 40% and 70% have been reported for antidepressant therapy in developed countries [4]. Non-adherence is associated with worse clinical and economic outcomes in observational studies [5,6].

There are no previous studies of adherence to antidepressants in Thailand. Only one retrospective study shows the pattern of prescriptions for antidepressants in 53 new cases of major depressive disorder in the out-patient psychiatric department of Siriraj hospital [7]. In Thailand, most general practitioners are not confident with the diagnosis of mental health conditions including major depression. The majority of depressive patients are treated in psychiatric hospitals and treatment coverage is low. According to an estimate from the Health Information Technology Center of the Department of Mental Health in Thailand only 3.4% of depressive patients in 2005 received treatment from the Ministry of Public Health including psychiatric hospitals and general hospitals [8]. The purposes of this study are to measure adherence to antidepressants and to determine the pattern of antidepressant prescriptions for treatment of major depression in a psychiatric institute in Thailand.

## Methods

### Data Source

This is a retrospective study using an electronic pharmacy data set which contains demographic, diagnostic, appointment and pharmacy information of outpatients in Galyarajanagarindra Institute, a psychiatric hospital in Thailand. The study protocol was approved by the Hospital Ethical Committee.

### Study Population

Patients were eligible for inclusion in the study if they were aged 15 and above and were newly diagnosed with major depression using International Classification of Diseases, Tenth Edition (ICD-10) codes as F32 (depressive episode), F33 (recurrent depressive disorder), and F38 (other mood/affective disorders)and F39 (unspecified mood/affective disorders). Inclusion into the study required patients to have received at least one prescription in these groups of antidepressants, namely tricyclic antidepressants -TCAs (amitryptyline, nortryptyline and imipramine), selective serotonin reuptake inhibitors -SSRIs (fluoxetine, escitalopram, fluvoxamine, paroxitine and sertaline) and other groups of antidepressants (tianeptine, trazodone and venlafaxine).

### Study Period

All patients were treated through the outpatient department between 15^th ^August 2005 and 29^th ^September 2008. The date of the first prescription for any of the antidepressants is defined as the index date. Data for individual patients were analyzed in the 6- month period following the index date. The 6-month timeframe was chosen to reflect the minimum time in which the American Psychiatric Association (APA) guidelines recommend patients be prescribed antidepressant therapy [9].

### Definitions and Measurement of Adherence

This study used a definition of adherence, taken from the conclusion reached by the participants at the World Health Organization (WHO) Adherence meeting in June 2001 is "the extent to which the patient follows medical instructions" [10]. There are many ways to measure medication adherence. However, none are considered the gold standard. Some suggest that the best way to measure adherence is comparing multiple methods [11].

Recent reviews [12-14] of adherence measures showed that the medication possession ratio (MPR) is a reliable measure of adherence. We utilized pharmacy records from databases to evaluate the MPR as a proxy for adherence to antidepressants [12].

MPR was defined using the continuous, multiple-interval medications available (CMA) methodology [15]. MPR is defined as the number of days for which the drug has been supplied during the follow-up period divided by the number of days elapsed during the period. From our dataset, the days of supply are calculated as dosage strength divided by daily dose and multiplied with the number of pills dispensed. For instance, a prescription for fluoxetine 40 mg/day, sixty 20-mg tablets, was calculated as (20/40) × 60 = 30 days' supply.

For patients who attended only once, we assume non-adherence as typical treatment should involve a 6-month course of antidepressants. Based on several studies on adherence measures in the psychiatric and medical literature, MPR < 0.8 represents non-adherence and 0.8≤ MPR≤ 1.0 represents adherence [12,16-18]. In the event that MPR was greater than 1.0, which reflected patients refilling antidepressants before the end of their medication supply or hoarding mediation for later use, the MPR value was truncated at 1.

## Results

There were 1,120 patients (6,025 visits) who were diagnosed with a depressive episode and received at least one antidepressant prescription. We excluded 62 patients who had missing age or were aged less than 15 years old. This left 1,058 eligible for study inclusion, 64% females and 36% males. Their average age was 46 with a range from 15 to 86 years. The majority of ICD-10 diagnostic codes for patients at first prescription were F32 -depressive episode (96.9%). There were few F38 and F39 (unspecified and other mood disorder) diagnostic codes (Table 1).

**Table 1:** Distribution of depression diagnosis of patients at first prescription

Diagnosis	Patients (%) (N = 1,058)
Depressive episode (not otherwise specified): F32, F32.8 and F32.9	34.1
Mild depressive episode: F32.0	5.8
Moderate depressive episode: F32.1	14.6
Severe depressive episode: F32.2 and F32.3	42.4
Recurrent depressive disorder: F33	0.0
Unspecified and other mood disorder: F38 and F39	3.1

Two thirds of patients were prescribed fluoxetine (Table 2). TCAs were the next most commonly prescribed class of drugs, followed by other drugs and other SSRIs.

**Table 2:** Percentage of patients ever prescribed each drug type

Drug group	Percentage
1. TCAs (amitryptyline, imipramine and nortryptyline, mianserin and mirtazapine)	43.8
2. Fluoxetine	67.4
3. Other SSRIs (escitalopram, fluvoxamine, paroxitine and sertaline)	23.1
4. Others (tianeptine, trazodone and venlafaxine)	39.3

* Percentages add to greater than 100% as some patients received two antidepressants concurrently or shifted from one drug to another during the follow-up periods.

Over the six-month period only 23% of patients (243 of the 1,058 cases) qualify as being adherent with a MPR greater than 0.80. Excluding the 470 patients who attended once only (we do not know if they continued to receive treatment elsewhere) 41% of patients were adherent and the overall MPR for those visiting more than once was 0.66 (Table 3). One-third of these patients received only one type of drug over the six month follow-up period and 30% were adherent.

**Table 3:** Adherence to any antidepressants at 6 months across patterns of prescriptions

Pattern of prescriptions	Cases	%Adherent (95% CI)	MPR	
			
			mean	SE
1. Received only one drug	195	30 (24-36)	0.57	0.02
2. Ever received 2 drugs at the same date	130	62 (54-70)	0.83	0.02
3. Switched from initial drug to a different one	263	39 (34-45)	0.63	0.02

* Patients qualify as being adherent if MPR is greater than 0.80

Adherence in the 22% of patients who received two drugs during the same visit was 62% and in the 45% of patients who were switched from one drug to another adherence was 39%.

## Discussion and Conclusion

Our study was a retrospective analysis of pharmacy data. The major strength of this form of analysis is that data arise from a real life setting rather than clinical trials. This is the first study to provide information on adherence to antidepressants in Thailand and it indicates that non-adherence is a problem for effective treatment of major depression in Thailand.

Numerous direct and indirect methods for measuring medication adherence are now available. MPR is an established method used in the assessment of medication adherence in pharmacy data analyses. It is non-invasive, easy to use and allows large numbers of patient records to be examined [19]. The MPR is considered a reasonable screening tool to determine patients with poor adherence that may benefit from interventions that aim to improve medication adherence [20].

A study of methods for evaluating patient adherence to antidepressant therapy found no significant difference in rates of 6-month antidepressant adherence between three methods the MPR, length of therapy (LOT) and combined MPR/LOT[12]. In addition, the MPR is a proxy measure of adherence that is widely used in retrospective data analyses [13,14]. Hence, we used MPR in our study.

The adherence in our study among patients attending at least twice is similar to the MPR results from a national database including data from patients who participated in 30 different health plans reported in US studies [12,21]. According to mental health experts in Thailand, the majority of cases are treated by psychiatric services with only a few patients being treated in primary care. We do not know how many of the 44% of patients who attended only once got further drug supplies elsewhere but it is likely that many of them did not. This means that the lower estimate of 23% adherence is a more likely estimate than the 41% based on more regular visitors. That would put adherence in Thailand at quite a lower level than reported elsewhere.

Given the large proportion of patients who switch between drug types, or are on multiple drug types, we cannot calculate adherence for individual drugs. As has been reported before, SSRIs are generally more tolerated than TCAs, but evidence has been conflicting [22]. One meta-analysis found a higher dropout rate for TCAs compared with SSRIs [23], whereas another showed no significant difference in the discontinuation rate between SSRIs and TCAs [24]. Recently, there has been contrasting evidence whether there is a difference in tolerability between those antidepressants.

The pattern of antidepressant prescribing for major depressive disorder is comparable to that found in an out-patient psychiatric department of a university affiliated hospital (Siriraj hospital) in Thailand [7]. That study also showed greater use of SSRIs or new generation antidepressants than TCAs. The proportion of patients who received multiple antidepressants was similar to a previous Thai study, with 23% receiving both TCAs and SSRIs in the previous study and 22% receiving multiple drugs in our study.

There is controversy surrounding the use of combination antidepressant treatments. Proponents believe there are combination medication options that are appropriate for patients suffering treatment-resistant depression (TRD) [25]. Opponents debate for possible toxicity and drug interaction consequences. A survey in Australia showed that nearly 80% of psychiatrists combine antidepressants [26]. However, 17% of respondents reported serious complications from combination antidepressant use such as epileptic seizures, hypomania and serotonin syndrome.

According to experts in Thailand, combination antidepressant therapy is commonly used by specialists. They would prefer to use a low dose of another antidepressant which has a sedative effect such as TCAs (amitryptyline) combined with SSRIs (fluoxetine) over the use of benzodiazepine for treatment of insomnia in major depressive patients.

There are limitations in this study that should be addressed. Firstly, several unverified assumptions potentially limit the interpretation of adherence by using the medication possession ratio, i.e. that 1) patients are actually taking drugs every time they refill their medications 2) patients do not receive medication outside the hospital pharmacy network; and 3) the MPR threshold of 0.8 is a valid threshold for adherence. In other words, according to those assumptions, the MPR can be overestimated if patients received drugs but not take them or it can be underestimated if they received antidepressants from other hospitals. As mentioned previously, most non-adherent patients in our study received only one prescription in this hospital and we do not know if these patients received subsequent prescriptions at other facilities. For this reason these patients were excluded from the MPR calculation.

Secondly, the results should be interpreted with the knowledge that medical adherence consists of both persistence (time to continued prescription) and compliance (obedience to follow the prescribed medication) [12]. However, the MPR should be interpreted with caution, since this ratio provides insight into medication adherence in terms of the proportion of time that the patients had possession of drug, but no indication as to the patterns of consistency of refilling. For example, in patients who get the same MPR some might be more consistent with refilling than others [27].

Lastly, there might be issues of generalisability as this study was conducted based on data from only a psychiatric hospital and results may not be comparable to those of patients in general hospitals.

Despite these limitations, non-adherence to antidepressant therapy is a problem in the management of depression in Thailand. Our study is an early step in establishing the MPR as a clinically useful way to estimate adherence among individual patients. As we know, factors that may affect adherence to medication fall into several categories related to medication, patient, doctor and other factors. The factors related to medication treatment include number of medications taken and side effects. The patient-related factors are educational background, cognitive impairment, co-morbidities, personal beliefs, patient personality and psychosocial profile. The doctor-related factors include doctor-patient relationship including doctor-patient communication. The examples for miscellaneous factors are healthcare access and social support. Future qualitative research could focus on the reasons for non-adherence and investigate reasons why people only attend once. Such studies would allow for a more accurate assessment of patient adherence.

## Abbreviations

CMA : Continuous, multiple-interval medications availability; MPR :   Medication possession ratio; SE : Standard error;  LOT: Length of therapy; DALYs : Disability Adjusted Life Years; SSRIs: Selective serotonin reuptake inhibitors; TCAs : Tricyclic antidepressants; TRD: Treatment-resistant depression

## Competing interests

The authors declare that they have no competing interests.

## Authors' contributions

BP conceived the study, designed the protocol, analyzed the data and prepared the manuscript. TV, PB and NC participated in the study design and significant comments on the manuscript. MB participated in the study design and helped to draft the manuscript. All authors have read and approved the final version of the manuscript.

## Pre-publication history

The pre-publication history for this paper can be accessed here:

http://www.biomedcentral.com/1471-244X/10/64/prepub
